# A Self-Assessment Stereo Capture Model Applicable to the Internet of Things

**DOI:** 10.3390/s150820925

**Published:** 2015-08-21

**Authors:** Yancong Lin, Jiachen Yang, Zhihan Lv, Wei Wei, Houbing Song

**Affiliations:** 1 School of Electronic Information Engineering, Tianjin University, 92 Weijin Road, Tianjin 300072, China; E-Mails: yclin@tju.edu.cn (Y.L.); yangjiac@163.com (J.Y.); 2 Shenzhen Institute of Advanced Technology, Chinese Academy of Sciences, 1068 Xueyuan Avenue, Shenzhen University Town, Shenzhen 518055, China; E-Mail: zh.lv@siat.ac.cn; 3 School of Computer Science and Engineering, Xi’an University of Technology, Xi’an 710048, China; E-Mail: weiwei@xaut.edu.cn; 4 Department of Electrical and Computer Engineering, West Virginia University, Montgomery, WV 25136, USA

**Keywords:** Internet of Things, information acquisition, stereo capture, self-assessment, long-distance shooting, quality assessment

## Abstract

The realization of the Internet of Things greatly depends on the information communication among physical terminal devices and informationalized platforms, such as smart sensors, embedded systems and intelligent networks. Playing an important role in information acquisition, sensors for stereo capture have gained extensive attention in various fields. In this paper, we concentrate on promoting such sensors in an intelligent system with self-assessment capability to deal with the distortion and impairment in long-distance shooting applications. The core design is the establishment of the objective evaluation criteria that can reliably predict shooting quality with different camera configurations. Two types of stereo capture systems—toed-in camera configuration and parallel camera configuration—are taken into consideration respectively. The experimental results show that the proposed evaluation criteria can effectively predict the visual perception of stereo capture quality for long-distance shooting.

## 1. Introduction

Nowadays, the rapid development of the Internet of Things (IoT) has aroused an increasing interest in various fields. Billions of people around the world use the Internet for browsing the web, sending and receiving emails, accessing multimedia content and services, playing games, using social networking applications and many other tasks [1,2]. It is predicted that IoT will change the way of people’s lives, promote millions of industrial developments and become a new driving force of informatization development. On the one hand, more and more people link to each other with access to such a global informationalized network. On the other hand, a global platform for intelligent machines and smart objects to communicate with each other is coming into reality. To develop the full potential of the IoT, great efforts have been devoted to solving serious technical challenges, such as data acquisition, identification of things, sensor network technologies, and so on [3,4,5,6,7]. However, how to get fully-reliable information data and to accurately predict their quality is still worthy of attention, since unqualified information will lead to unreasonable results.

Serving as one of the major carriers of information, stereo capturing-based contents have been widely used in IoT, such as web conferencing, 3D gaming, object tracking, face detection and tracking, automotive safety, cultural heritage object modeling, robotics and medical devices [8,9,10,11,12]. In the industrial fields, 3D technology has been applied to process control, industrial detection, computer-aided design and manufacturing (CAD/CAM) and remote monitoring, which leads to unprecedented visual realism. In the architecture fields, stereoscopic images help experts and engineers examine structural information, display design, decoration, landscaping and other aspects. All of the applications can be devoted to the intelligent industry and smart homes with the help of IoT. Therefore, the question becomes: how does one make sure that the stereoscopic contents have higher quality and better 3D perception, under the circumstances of various distortions and impairments in real applications? Stereo capture is the major procedure to get stereoscopic pictures, and the application of stereo capture has gained significant growth in recent years. Based on the investigation of 3D perception, the major concern of stereo capture is to set the stereo camera configuration by choosing an appropriate focal length, shooting distance, inter-camera distance and other parameters [13]. Even a slight change in these parameters often causes spatial distortions in stereoscopic image pairs, such as the puppet-theater effect and the cardboard effect [14,15]. These distortions may cause human visual discomfort and poor 3D effects [16,17,18,19] when people are watching stereoscopic images displayed on 3D displays. In this paper, we come up with a stereo capture system with self-assessment capability to minimize the impact of distortion and impairment in long-distance shooting applications and try to develop the sensor for stereo capture in a more intelligent mechanism, which would promote the utilization of IoT sensors.

Against this background, Section 2 introduces related work, and Section 3 presents the subjective quality assessment experiment on stereoscopic contents, followed by a detailed description of the evaluation criteria for stereo capture in Section 4. Furthermore, Section 5 describes the evaluation experiments. Finally, Section 6 concludes the paper.

## 2. Related Work

Currently, researchers have focused on the analysis of shooting principles to get an excellent stereo effect; for instance, Frederik *et al.* presented production rules that were required for the acquisition of adequate stereo content through the study of the geometry of the 3D display, 3D capture and the stereo formula [10]; Kim *et al.* reported a visual fatigue metric that could replace subjective tests to evaluate image quality; further, the metric could also be used for filming and warning systems for general viewers [16]; Kim and Sohn proposed a 3D reconstruction algorithm from a stereoscopic image pair through analyzing inter-camera distance, camera focal length and shooting distance; this method solved the mutual occlusion and interaction problems between real and virtual objects in an MRsystem [20]; Lee *et al.* used a multiple color-filter aperture camera to present an adaptive background generation method for automatic selection of initial object regions, which can be appropriate for realizing depth capture and simultaneous detection [21]; Min *et al.* present a new method of synthesizing novel views from the virtual cameras in multiview camera configurations for 3DTV system [22]; Yamanoue, Okui and Yuyama presented a setting principle to achieve better stereo quality through analyzing the relationship between camera focal length and the convergence point [23]. Additionally, many scholars have exerted their efforts to establish systems for stereo capture, such as: Ebrahimnezhad *et al.*, who constructed a set of calibrated virtual stereo cameras to propose a robust curve-based method for 3D model reconstruction of an object from image sequences captured by perpendicular stereo rigs [24]; Heinzle presented a computational stereo camera system that could close the control loop from the capture and to analyze the automatic adjustment of physical camera parameters [25]; Ilham *et al.* established a semi-automatic camera rig system equipped with inter-camera distance and the convergence angle [26]; Jung *et al.* equipped stereo vision cameras with a time-of-flight camera to propose a novel visual discomfort monitoring, and the stereo-plus-depth camera system had a left view color camera, a right view color camera to capture the stereoscopic images and a time-of-flight camera to capture the depth map [27]; Lim *et al.* proposed a simple geometrical ray approach to calibrate the extrinsic parameters of the cameras and solved the stereo correspondence problem of the single-lens bi-prism stereovision system [28]; Oskam *et al.* presented a controller system for camera convergence and inter-axial separation that specifically addressed challenges in interactive stereoscopic applications like games [14]. Furthermore, Okui, Hanning, Zhu and Park *et al.* had done some related studies about the effect of camera parameters on shooting quality [15,29,30,31].

Although stereo capture technologies have made significant progress, most studies were about part of the stereo camera parameters. The stereo capture systems were built mainly for specialized cameras or hardware platforms, and the widely-recognized objective stereo cameras shooting quality evaluation criteria have not been fully investigated yet. At the same time, some subjective evaluation theories were proposed to assess the quality of stereo images [15,32,33]; however, the subject evaluation required numerous duplicate tests with a great number of participants. It is a time-consuming process for stereo capture, and no immediate feedback is able to provide for the resetting of stereo cameras, if the shooting quality is not as ideal as expected. For these reasons, the shooting parameters of toed-in and parallel stereo camera configurations, as well as the basic shooting principles are fully investigated, in order to establish an objective shooting quality evaluation criteria applicable to stereo capture systems for long-distance shooting. The evaluation criteria provide the design of integrated stereo camera systems with a basic indication of shooting quality, which can be regarded as the basis for the self-assessment capability. When the self-assessment capability gives lower shooting quality, an adjustment procedure of shooting parameters is required until the evaluation criteria reach a reasonable value and, therefore, a better 3D effect in accordance to people’s subjective perception appears.

## 3. Subjective Quality Assessment Experiment on Stereoscopic Contents

Different from the traditional 2D subjective quality assessment method, an additional indicator, such as depth rendering, naturalness, presence, visual experience, visual comfort, *etc.*, should be taken into consideration in 3D assessment [34]. In this section, subjective quality assessment based on the 3D evaluation concept is used to evaluate the generated stereoscopic contents in order to justify the proposed optimal shooting rules.

**Figure 1:**
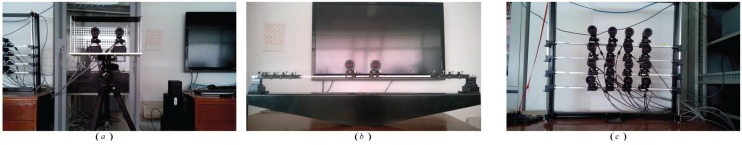
Real stereo camera schematic diagram. SONY ICX445 CCD, 1/3″, 3.75 *µ*m; Global Shutter, 1024 × 768 at 30 FPS. (**a**) Inter-camera distance can be changed to obtain stereo images with different shooting distances, and also, the toed-in and parallel camera configurations can be obtained; (**b**) bigger inter-camera distance; (**c**) matrix multi-camera arrangement.

(1) Participants: Fifty non-professional adult assessors, aged between 20 and 40 years with a binocular vision of more than 0.8, participated in the subjective assessment. All participants had normal stereoacuity according to the Titmus stereo test.

(2) Stereo images: Double viewpoint images were adopted for the subjective experiments. These images were selected from the stereo image library in the stereo vision laboratory of the School of Electronic Information Engineering, Tianjin University [35]. The main sources of stereo images in this library are captured by Autodesk 3ds Max and stereo cameras in the laboratory (shown in Figure 1a–c). The resolution of the training and test stereo images is 1024 × 768. This database consists of 1839 stereoscopic pairs (990 for the toed-in camera configuration and 849 for the parallel camera configuration) under various shooting camera parameters.

(3) Display setting: The current research is motivated by the need to enhance the understanding of the variables that may influence the shooting quality of stereoscopic images. Since the display is the most common media through which people watch stereoscopic images and perceive the shooting quality, thus several display aspects, e.g., display size and watching condition, should not be overlooked [16]. It is important to acknowledge that the depth that is perceived in stereoscopic contents is strongly related to the size of the display screen [32]. Subjective tests in this paper are conducted on two different sizes of stereoscopic displays, namely the Hyundai S465D 46-inch 3D stereoscopic LCD display and the LG 47CM540-CA 47-inch 3D HDTV display, to build shooting principles. The two are paired with their own 3D active glasses.

(4) Procedure: Before formal experiments, all participants watched randomly-ordered training stereo images for 8 s at a distance of approximately 3.9 m, as suggested in the ITU-RBT.1438 for HDTV [36]. They were then asked to evaluate the stereo images with different camera parameters at the viewing range suggested by the instructions for each display. During the subjective tests, a series of stereo images under the guidance of the long-distance shooting principle, which were captured with a common value of shooting distance *h* and changing values of other camera shooting parameters, was displayed for 5 s, followed by a 5-s interval of a 2D mid-gray image as a grading and relaxation period. For each of the durations, observers were asked to rate the quality of stereo images using a five-level scale, as shown in Table 1.

The mean opinion score (MOS) [37] was firstly computed for each image by averaging all of the subjective scores. Then, we calculated the range of each influenced factor, which is further introduced in Section 4, and summarized the relationship between each factor and the MOS values. The experimental processes were repeated with the changing of the value of *h*. At last, we presented the total evaluation factor for toed-in and parallel camera configurations, respectively.

**Table 1:** Standards for the subjective quality evaluation of stereo cameras.

Response	Explanation	Quality
	**Imperceptible**: Not any damage to	
5	3D or image quality, looks comfortable and	Excellent
	natural, consistent with human visual experience.	
	**Perceptible, but not annoying**: A slight loss of	
4	3D or depth perception, but the quality of the whole	Good
	image is still good, consistent with human visual experience.	
	**Slightly annoying**: Obvious loss of 3D and depth	
3	perception; however, you can accept viewing such quality,	Fair
	reluctantly, and it is generally suitable for human visual experience.	
2	**Annoying**: Need to attentively distinguish between 3D and	Poor
	depth perception that is not suitable for visual experience.	
1	**Very annoying**: Nearly no 3D perception, and	Bad
	people feel uncomfortable.	

## 4. Evaluation Criteria for Stereo Capture

**Figure 2:**
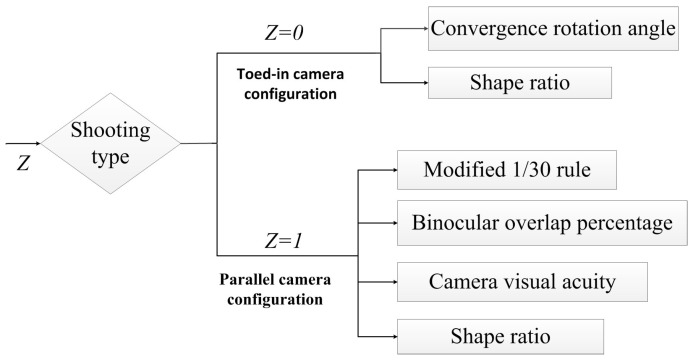
Objective stereo cameras’ shooting quality evaluation criteria for long distances.

Stereo cameras are generally divided into two types in shooting configurations: toed-in and parallel [15,38]. By analyzing the features of the toed-in and parallel camera configurations, we found that different parameter settings had significant influence on the 3D effect of stereoscopic image pairs. In addition, we found that the image quality was still good when the evaluation factor exceed the previous shooting guidelines. The previous shooting guidelines were empirical methods and could only generate a rough estimation and suggestion for the camera parameters. Therefore, this paper aims to establish a corresponding five-level evaluation criteria for stereo camera configurations over long-distance shooting, as shown in Figure 2. The semantic meaning of stereo camera parameters used in establishing the evaluation criteria is summarized in Table 2.

**Table 2:** Meaning of the stereo camera parameters.

Camera Parameters	Semantic Meaning
*h*	the shooting distance
*d*	the inter-camera distance
*f*	the camera focal length
*p*	the viewing angle

### 4.1. Evaluation Criterion for the Toed-In Camera Configuration

For the toed-in camera configuration, the optical axes are converging on a single point. The objects in the foreground have a significant effect on the stereo quality of the images. Convergence rotation angle *α* [25] and shape ratio *μ* [10] are important factors that have to be taken into consideration for the toed-in camera configuration over long-distance shooting.

**Figure 3:**
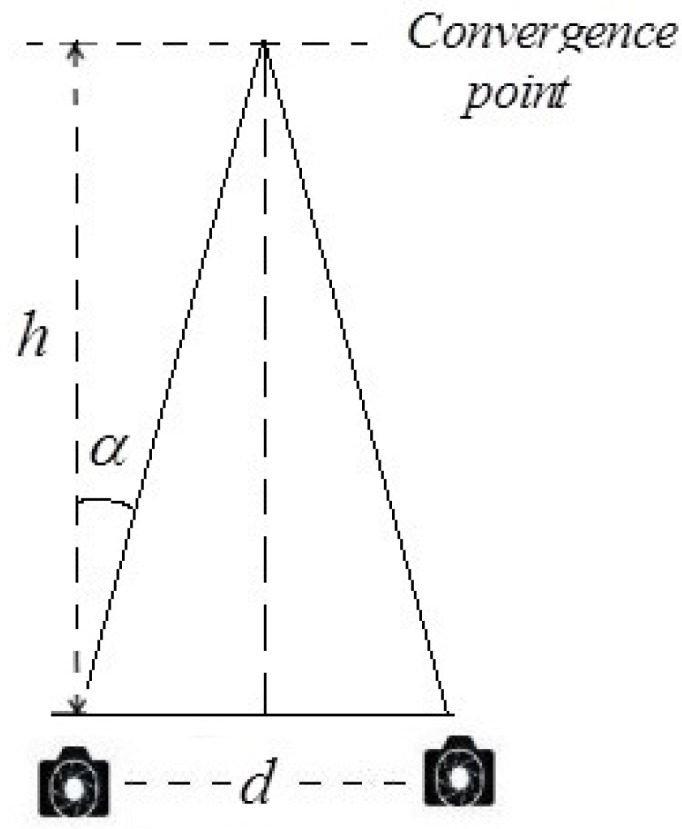
Computation of screen space disparities.

Convergence rotation angle: Heinzle *et al.* [25] presented that the value of convergence rotation angle *α* (Equation (1)) has an effect on the position of convergence point, as shown in Figure 3.

(1) $$\alpha =arctan(\frac{d}{2\cdot h})$$

Previous studies indicated that *α* had an effect on the screen disparity of a given point, while it referred to the distance between the two corresponding points in stereoscopic images recorded by the left and right cameras. The disparity was often the most important parameter for stereo depth perception and related to the most comfort-zone constraints. However, it was an empirical method and could only generate a rough estimation for camera parameters. Hence, this part of the whole aims to establish a corresponding five-level evaluation criterion of the convergence rotation angle for stereo cameras through subjective and objective experiments.

**Figure 4:**
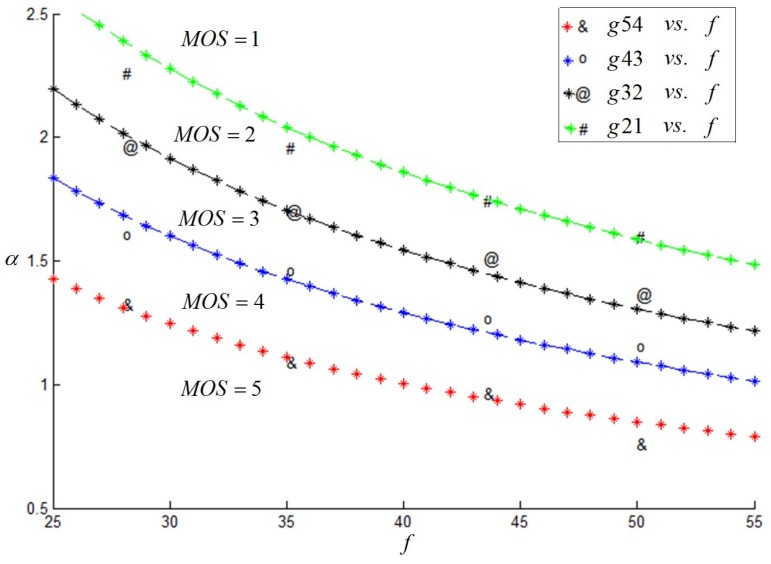
Variation trend of *α*(°) with *f* (mm).

As a matter of convenience, this paper considered *α* as the evaluation index. Firstly, when *f* = 50 mm and *h* was a fixed value, *d* changed constantly. Then, we change the value of *h*, and we captured the corresponding stereoscopic image pairs once again. Besides, we changed the camera focal length *f* and carried out similar experiments as described above. The subjective results are shown in Figure 4, and it was indicated that *f*, as well as the *α* value had a great effect on the subjective results.

In order to enrich the experiments, several of the camera focal lengths were involved in our experiments. Through a number of experiments, we proposed an ultimate assessment index *g*, calculated from Equation (2). The mapping between *g* and the MOS value, $MOS_{g}$, was studied based on the subjective experimental results and is listed in Table 3. It indicated that when the *g* value was at most 20.56, a good stereoscopic effect could be obtained.

(2) $$g=\alpha \cdot f^{0.7506}$$

**Table 3:** Mapping between *g* and the mean opinion score (MOS) value.

${\mathrm{MOS}}_{g}$	Index: *g*
5	*g*≤16.00
4	16.00<*g*≤20.56
3	20.56<*g*≤24.61
2	24.61<*g*≤25.09
1	*g*>25.09

Shape ratio: Frederik *et al.* [10] proposed that the shape ratio *μ* was defined as the depth magnification to the width magnification. Equation (3) can be taken to ensure an undistorted depth reproduction near the screen surface:

(3) $$\mu =\frac{h_{D}\cdot d}{h\cdot t_{e}}$$

Where $h_{D}$ is the viewing distance, $t_{e}$ is the viewer’s inter-ocular distance, *h* is the shooting distance and *d* is the inter-camera distance. Note that, if an average $h_{D}$ and the point of convergence are given, the grade of stereoscopic distortion can only be controlled by choosing the right ratio between *d* and $t_{e}$.

The geometrical interpretation of Equation (3) depended on whether the points at infinity were actually presented in the captured scene. The value of *μ* had a significant effect on the quality of the stereoscopic image pairs. The smaller the value, the higher the stereo quality. Hence, when shooting long distance, *h* was far outstrips $h_{D}$ and *d* was bigger than $t_{e}$. This part of the whole aims to establish a corresponding five-level evaluation criterion of the shape ratio for the stereo camera through experiments.

Based on the experiments, a series of stereoscopic image pairs was captured with different shape ratios. We selected a bigger viewing distance as 3.9 m; refer to [36] for HDTV. The range of *μ* is from 0.095 to 1.532. Through subjective experiments, we determined the mapping between *μ* and the MOS value, $MOS_{\mu t}$, of stereoscopic image pairs, shown in Table 4, which indicated that when the *μ* value was no more than 0.304, a good stereoscopic effect could be obtained.

**Table 4:** Mapping between *μ* and the MOS value.

${\mathrm{MOS}}_{\mu t}$	Index: *μ*
5	*μ* ≤ 0.235
4	0.235 < *μ* ≤ 0.304
3	0.304 < *μ* ≤ 0.361
2	0.361 < *μ* ≤ 0.623
1	*μ* > 0.623

### 4.2. Evaluation Criterion for Parallel Camera Configuration

For the parallel camera configuration, the evaluation of long-distance shooting quality has been studied from the following four aspects: 1/30 rule [39,40], binocular overlap percentage [15,30,41], camera visual acuity [42] and shape ratio [10].

Modified 1/30 rule: In professional stereo shooting activities, the 1/30 rule of 3D, which stipulates that the inter-camera distance *d* should be 1/30 of the shooting distance *h* from the camera to the first foreground object, was suggested and widely used in stereo photography. In our experiments, the index d/h was applied to the analysis of the effect on shooting quality.

Previous studies presented that the 1/30 rule was a two-level evaluation criterion, which meant two evaluation effects (good or bad) toward the stereo effect [39,40]. Therefore, our goals were complementary to these previous works. Our work targeted establishing the five-level objective evaluation criterion. As a matter of convenience, this paper considered d/h as the evaluation index. Based on experiments, a series of stereoscopic image pairs was captured, and the value of d/h ranged from 1/80–1/15.

Combining with the subjective experimental results and the range of the d/h value, the mapping between d/h and the MOS value, $MOS_{dh}$, was calculated, as shown in Table 5, and it indicated that when the d/h value was at most 1/39.685, people can obtain a good stereoscopic effect.

**Table 5:** Mapping between d/h and the MOS value.

${\mathrm{MOS}}_{\mathrm{dh}}$	Index: d/h
5	d/h ≤ 1/53.125
4	1/53.125 < d/h ≤ 1/39.685
3	1/39.685 < d/h ≤ 1/30.555
2	1/30.555 < d/h ≤ 1/26.420
1	d/h > 1/26.420

**Figure 5:**
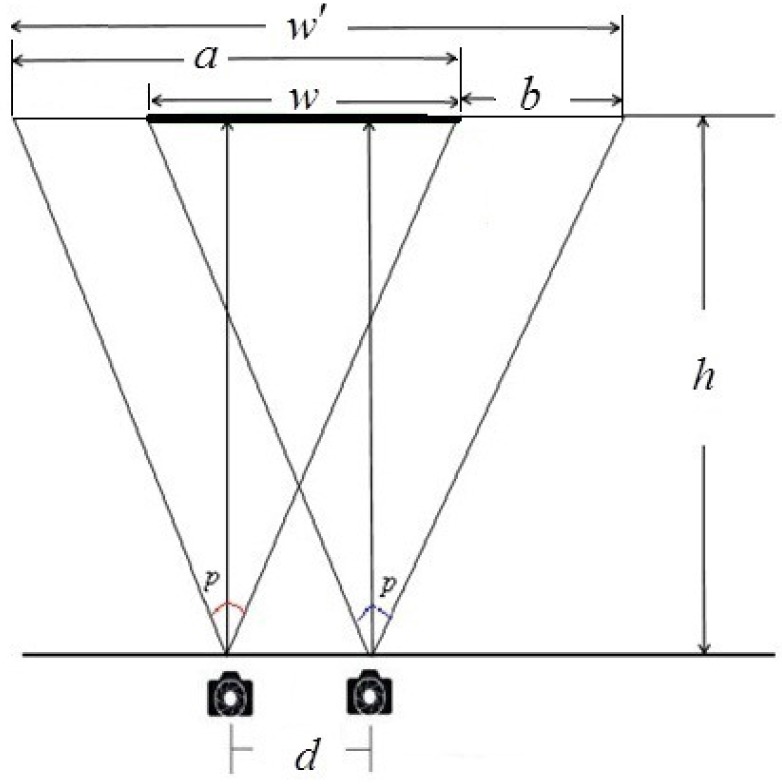
Schematic diagram of binocular overlap.

Binocular overlap percentage: The magnification of an image on the retina is a/w, as shown in Figure 5 (here, *a* is the original image width, *p* is the viewing angle of the stereo camera, $w^{\prime }$ is the viewing region of stereo camera and *w* is the resulting composite image width, which denotes the binocular overlap of stereo camera). a/w can influence the values of positive and negative parallax and further affect the quality of the stereo images. In order to simplify the calculation, $w/w^{\prime }$ was applied to the analysis on how binocular overlap affected stereoscopic capturing quality. Based on the geometric relations in Figure 5, we can conclude:

(4) $$\begin{array}{c} \left\{\begin{array}{c} b=d\\ \frac{w}{w^{\prime }}=\frac{w}{a+b}\\ h=\frac{a}{2\cdot \tan(p/2)} \end{array}\right. \end{array}$$

For the sake of better understanding, the principle of the binocular overlap percentage was taken as the evaluation index. All test stereo images were divided into several different groups, each with a fixed value of *h* and different values *d*. Then, changing the value of *h* and repeating the above experimental process, a series of stereoscopic image pairs was captured. Through subjective experiments, we took index *ξ* as the binocular overlap percentage $w/w^{\prime }$ in Equation (4) with the effort of Equation (5).

(5) $$\xi =\frac{w}{w^{\prime }}=\frac{2\cdot h\cdot tan(p/2)-d}{2\cdot h\cdot tan(p/2)+d}$$

Based on a series of experiments, the binocular overlap percentage *ξ* of stereoscopic image pairs can be calculated, and the range of *ξ* is from 0.706–0.995. Through investigating the mapping between *ξ* and the MOS value $MOS_{\xi }$, as shown in Table 6, it can be used to evaluate the effect of the binocular overlap for long-distance shooting with the parallel camera configuration.

**Table 6:** Mapping between *ξ* and the MOS value.

${\mathrm{MOS}}_{\xi }$	Index: *ξ*
5	*ξ*≥0.955
4	0.943≤*ξ*<0.955
3	0.927≤*ξ*<0.943
2	0.899≤*ξ*<0.927
1	*ξ*<0.899

**Figure 6:**
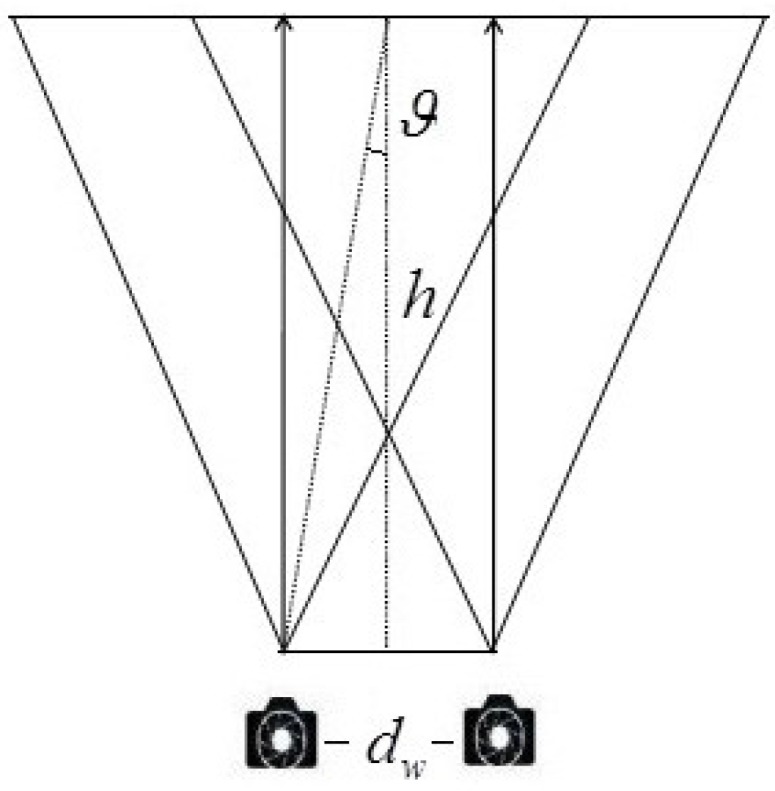
Schematic diagram of camera visual acuity.

Camera visual acuity: Generally, the camera visual acuity is widely recognized as $0.57^{\circ }$ (*ϑ*; shown in Figure 6). Let *h* denote the shooting distance; the theoretical inter-camera distance $d_{w}$ can be obtained (shown in Equation (6)) according to the camera visual acuity.

(6) $$tan\left(\vartheta \right)=\frac{d_{w}}{2\cdot h}$$

(7) $$\vartheta =arctan(\frac{d_{w}}{2\cdot h})$$

**Figure 7:**
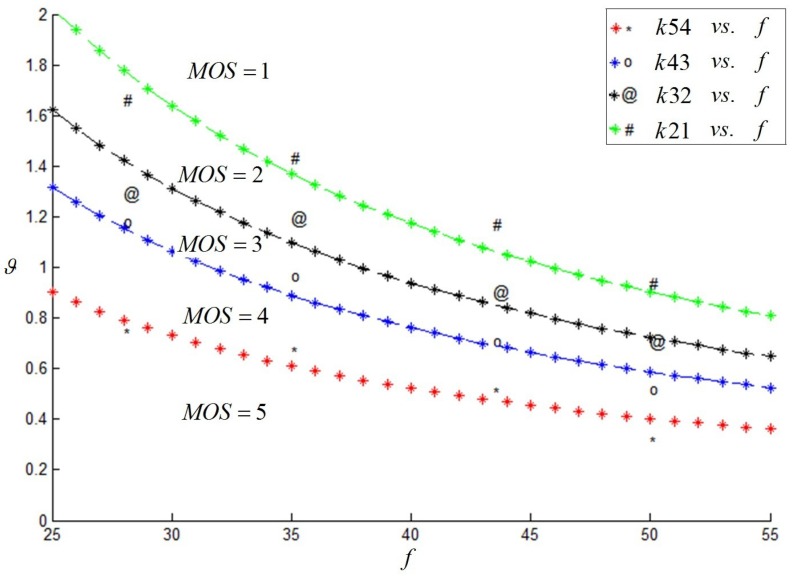
Variation trend of *ϑ*(°) along with *f* (mm).

Over the long-distance shooting condition, the camera visual acuity was one of the main considerations. Ignoring this limitation might result in viewing discomfort or even the loss of stereo impression. In order to establish a five-level evaluation criterion, this paper took *ϑ* (shown in Equation (7)) as the evaluation index of camera visual acuity.

In order to enrich the experiments, several of the camera focal lengths *f* were involved in our experiments; the experimental results are shown in Figure 7, which indicated that *f*, as well as *ϑ* have a great effect on the subjective results. We proposed an ultimate assessment index *k*, calculated from Equation (8). The mapping between *k* and the MOS value, $MOS_{k}$, is shown in Table 7. The values indicated that when *k* was at most 55.98, a good stereoscopic effect could be obtained.

(8) $$k=\vartheta \cdot f^{1.165}$$

**Table 7:** Mapping between *k* and the MOS value.

${MOS}_{k}$	Index: *k*
5	*k*≤38.41
4	38.41<*k*≤55.98
3	55.98<*k*≤60.00
2	60.00<*k*≤86.23
1	*k*>86.23

Shape ratio of the parallel camera configuration: Similarly, the shooting principle of the shape ratio was applied to the parallel camera configuration. Based on a series of experiments, the value of *μ* ranged from 0.095–0.932. Combined with the subjective experimental results, shown in Table 8, the mapping between *μ* and the MOS value, ${MOS}_{\mu p}$, of stereoscopic image pairs had been investigated.

**Table 8:** Mapping between *μ* and the MOS value.

${MOS}_{\mu p}$	Index: *μ*
5	*μ*≤0.120
4	0.120<*μ*≤0.147
3	0.147<*μ*≤0.201
2	0.201<*μ*≤0.226
1	*μ*>0.226

### 4.3. Comprehensive Objective Evaluation Criteria

At present, the most common method used to integrate all independent individual factors into a global index is the linear weighting method [43,44,45]. In order to reasonably evaluate the performance of the objective evaluation criteria, we applied a linear regression to the combination of the six factors, and each of them was given a weight. We specified $MOS_{g}$ as the output value of the convergence rotation angle for the toed-in camera configuration, $MOS_{\mu t}$ as the output value of the shape ratio factor for the toed-in camera configuration, $MOS_{dh}$ as the output value of the modified 1/30 rule factor for the parallel camera configuration, $MOS_{\xi }$ as the output value of the the binocular overlap percentage factor for the parallel camera configuration, $MOS_{k}$ as the output value of the camera visual acuity factor for the parallel camera configuration and $MOS_{\mu p}$ as the value of the shape ratio factor for the parallel camera configuration. Considering that each of the factors had different properties in stereo capture, the establishment of the evaluation criteria should take all of the perspectives into account. Accordingly, they were regarded as individual factors that could be considered relatively independent. Although there may be a relation among the criteria, for simplicity, the global quality score *Q* can be gained by using a linear combination of the criteria, which can be defined as:

(9) $$Q=\{\begin{array}{ll} m\cdot MOS_{g}+n\cdot MOS_{\mu t} & \text{Z=0}\\ q\cdot MOS_{dh}+r\cdot MOS_{\xi }+ & \text{Z=1}\\ s\cdot MOS_{k}+t\cdot MOS_{\mu p} & \end{array}$$

where Z=0 denotes toed-in camera long-distance shooting, Z=1 denotes parallel camera long-distance shooting, *m*, *n*, *q*, *r*, *s* and *t* are weights of each factor in objective evaluation criteria and restricted by m+n=1 and q+r+s+t=1.

With the given weights of the factors, we can compute an objective score for each captured 3D image pair. At the same time, through a series of subjective tests, which has been done in Section 2, the subjective score for each pair can also be obtained. In order to choose the proper values of the six weights, the correlation coefficients between the objective and subjective scores were computed on the whole database, and the corresponding values that achieved the max relativity were chosen for the weight of each factor. Based on the experimental results, the weights of each factor are shown in Table 9. It is worth noting that the factors for each camera configuration get the same weight, indicating that the criteria proposed may have approximately the same importance in evaluating the performance of 3D capturing. However, the application of 3D capturing is a still a complex procedure, and more efforts are need.

**Table 9:** Weights of each factor in the objective evaluation criteria.

**Shooting Type**	*m*	*n*	*q*	*r*	*s*	*t*
Z=0, toed-in	0.5	0.5	0	0	0	0
Z=1, parallel	0	0	0.25	0.25	0.25	0.25

## 5. Evaluation Experiments

To verify the proposed objective criteria, another thirty non-professional adults, aged between 20 and 40 years, participated in the subjective evaluation experiments. All of them took the stereo vision test before the subjective experiments. They were asked to watch the training stereo images with different camera parameters. Two hundred and nineteen test stereoscopic image pairs were used for the subjective test and displayed randomly.

**Figure 8:**
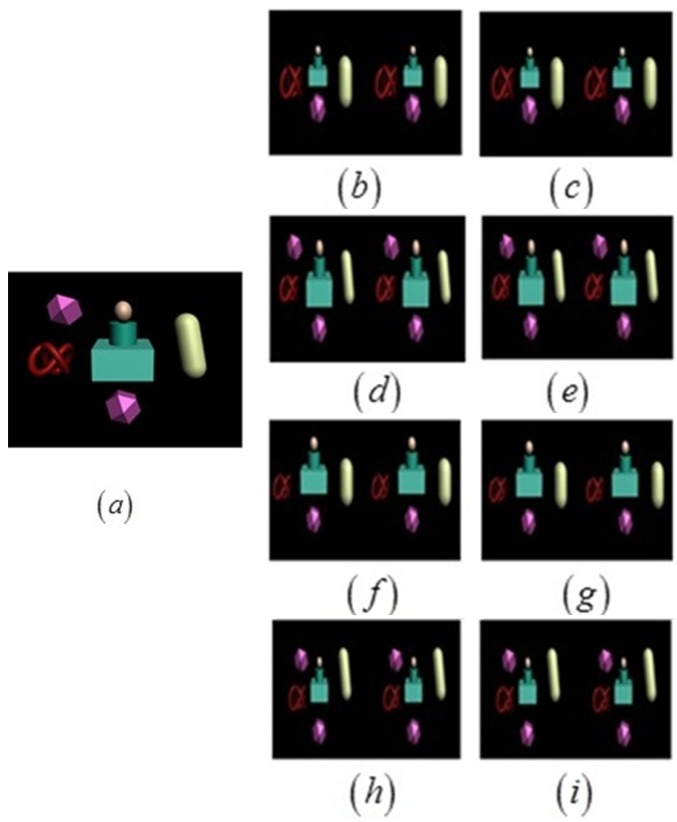
Stereoscopic image pairs captured by toed-in and parallel camera configuration long-distance shooting. (**a**) The selected scene; (**b**–**e**) Captured by the toed-in camera configuration; (**f**,**i**) Captured by the parallel camera configuration.

One of the selected scenes is shown in Figure 8a. Under the condition of the long-distance shooting setting, stereoscopic image pairs were captured by changing the value of the main camera parameters *h* and *d*, when *f* = 50 mm, using toed-in and parallel camera configurations, respectively.

Take the stereoscopic image pairs captured by the parallel camera configuration as an example, shown in Figure 8b–e. When *d* = 600 mm, *h* = 50 m, the quality score *Q* is 5.0. Learning from the subjective experiments, the MOS is also 5.0, which indicates that the prediction of the proposed criteria is consistent with the subjective evaluation value. When *d* = 1800 mm, *h* = 50 m, *Q* is 2.0. Based on the subjective experiments, the MOS is 2.3, which indicates that our proposed criteria are in line with human perception. According to our evaluation formula, *Q* will decrease as the value of *d* increases. When *d* = 2400 mm, *h* = 90 m, *Q* is three and the MOS is 3.3, which is close to the value from the objective value.

Stereoscopic images captured by the toed-in camera configuration are shown in Figure 8f–i. When *d* = 1000 mm, *h* = 70 m, *Q* is 5.0. Learning from the subjective experiments, the MOS is 5.0, which is in line with the output of our proposed criteria. When *d* = 3000 mm, *h* = 70 m, *Q* is 3.5 and the MOS is 3.8. The comparison between the two results reveals that the value of *d* has a great effect on stereo image quality, which is consistent with our prediction. Similar results can also be found on other captured images.

**Figure 9:**
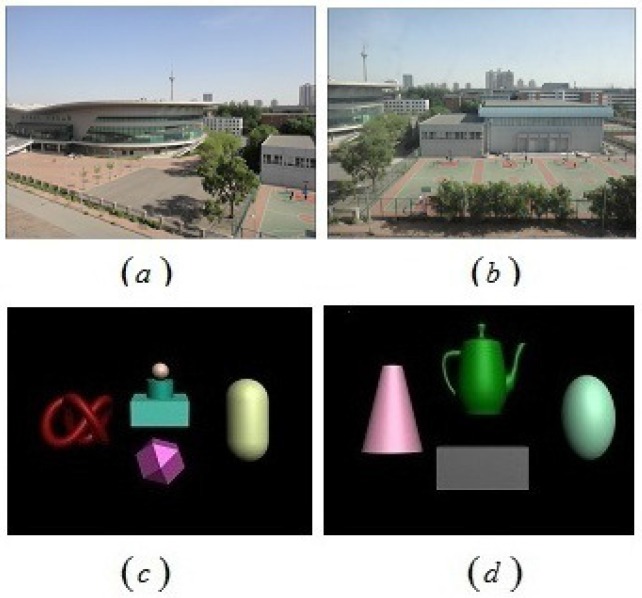
Left view of the stereoscopic image pairs from the real camera and Autodesk 3ds Max shooting. (**a**,**c**) Long-distance shooting with the toed-in camera configuration; (**b**,**d**) Long-distance shooting with the parallel camera configuration.

**Figure 10:**
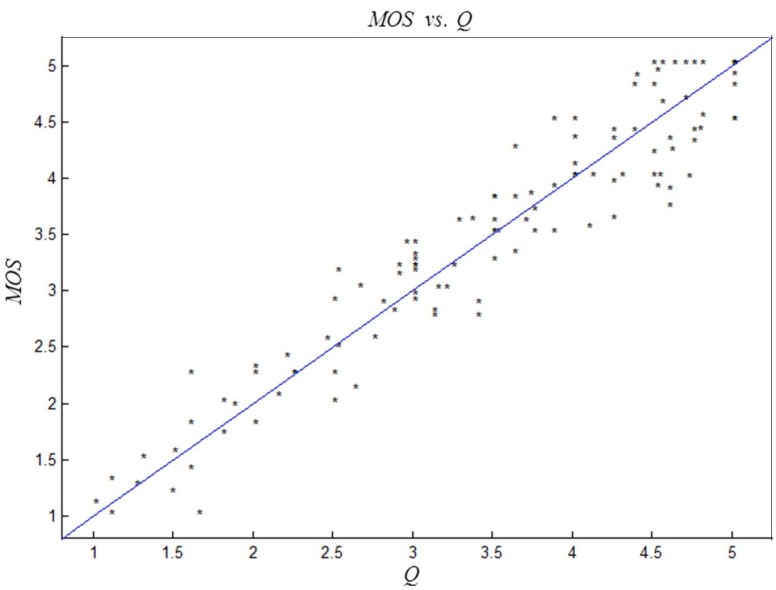
Schematic diagram of the correlation between quality score *Q* and subjective values MOS.

In order to further validate the effectiveness of the proposed objective evaluation criteria, another four group scenes were chosen to conduct the above experiments. Two groups were real 3D scene pictures (shown in Figure 9a,b), and the rest were Autodesk 3ds Max scene pictures (shown in Figure 9c,d). Through changing the values of shooting parameters *h*, *d* and *f*, another two hundred and sixty-one stereoscopic image pairs were chosen to validate the effectiveness of the proposed criteria. The linear correlation between objective evaluation criteria *Q* and the subjective evaluation MOS values is shown in Figure 10. As we can see from the figure, the consistency between the proposed criteria and the subjective evaluation is clearly identified.

Three evaluation indices are adopted to verify the consistency of the objective evaluation criteria and the subjective evaluation values for long-distance stereo capture quality [46]: the Pearson correlation coefficients (CC), the Spearman rank order correlation coefficient (SROCC), and the RMSE. The range of CC and SROCC is 0–1; the closer the values of CC and SROCC are to one, the better the performance of the criteria, and *vice versa*. The range of RMSE values is 0 to +∞, and the lower its value, the better the performance of the criteria. Thirty image pairs were selected from the two hundred and sixty-one test stereoscopic image pairs to compare the output values of subjective and objective evaluation criteria, and the results are summarized in Table 10.

According to Figure 10 and Table 10, we can conclude that the objective evaluation results of the proposed criteria are in accordance with those of the subjective evaluation. With the verification of subjective experimental results and theoretical analysis, the proposed criteria algorithm is applicable for evaluating the long-distance shooting quality of stereo cameras.

**Table 10:** MOS, *Q*, Spearman rank order correlation coefficient (SROCC), correlation coefficients (CC) and RMSE of the selected thirty stereoscopic image pairs.

Image	MOS	*Q*	SROCC	CC	RMSE
1	5.0	5.0	1.0000	0.9995	4.6438
2	3.3	3.0	0.9974	0.9983	5.9059
3	5.0	5.0	1.0000	0.9985	4.2040
4	2.3	2.0	0.9974	0.9718	5.8688
5	5.0	5.0	1.0000	0.9416	2.7812
6	3.8	3.5	0.9974	0.9118	3.9980
7	5.0	5.0	1.00000	0.9211	2.8320
8	4.0	4.5	0.9929	0.9803	3.6014
9	3.25	3.0	0.9982	0.9885	5.5181
10	4.0	4.75	0.9942	0.9919	5.2442
11	4.0	4.0	1.0000	0.9781	4.4100
12	2.25	2.0	0.9982	0.9786	4.9968
13	4.2	4.5	0.9974	0.9527	4.6665
14	3.7	3.75	0.9999	0.9746	5.0058
15	5.0	4.75	0.9982	0.9848	4.5051
16	3.2	3.0	0.9989	0.09808	5.1612
17	3.5	3.5	1.0000	0.9762	5.6040
18	4.0	3.5	0.9974	0.9785	5.1769
19	4.33	4.0	0.9969	0.9713	3.6109
20	2.25	2.5	0.9982	0.9748	3.9698
21	5.0	5.0	1.0000	0.9583	3.2402
22	4.8	4.5	0.9974	0.9532	3.2718
23	4.5	4.0	0.9929	0.9608	3.6055
24	3.2	3.0	0.9989	0.9671	4.0700
25	4.0	4.0	1.0000	0.9946	3.5376
26	3.0	3.2	0.9989	0.9889	3.9217
27	2.0	2.5	0.9929	0.9892	4.0989
28	1.8	2.0	0.9989	0.9888	4.5445
29	5.0	5.0	1.0000	0.9526	3.6583
30	4.5	5.0	0.9929	0.9626	3.1121

## 6. Conclusions

In this paper, we proposed the design of a self-assessment stereo capture model applicable to IoT for long-distance shooting. Serving as a major procedure in information acquisition, stereo capture plays a key role in determining the quality of stereo capturing-based content in IoT. Regarded as the core component of the self-assessment model, an objective evaluation criteria for long-distance shooting quality adapted to different stereo camera configures is fully investigated. Two types of stereo camera systems—toed-in camera configuration and parallel camera configuration—were taken into consideration respectively. Experimental results indicated that the proposed evaluation criteria were consistent with people’s subjective perception and can be applied to the self-assessment of the stereo capture’s long-distance shooting quality. Instead of duplicate subjective tests, the design of the self-assessment capability can effectively predict the long-distance shooting quality and provides a better 3D effect in accordance to people’s subjective perception in a much easier way. In conclusion, the establishment of the evaluation criteria will not only lead to better shooting quality, but also provide a theoretical basis for the integrated stereo capture systems with self-assessment capability. With this model, more reliable stereo image-based information can be used in the interaction between physical terminal devices, and the IoT will also benefit a lot from improved sensor networks.

It is worth noting that a self-assessment stereo capture model that can compute its performance by using the provided criteria has been realized in the paper. However, there is still a lack of research on how to improve the performance. Further studies and analysis will focus more on the design of the stereo capturing system with the ability to adaptively adjust corresponding parameters to achieve better capture quality, if the system has only poor performance.

## Appendix

**Meaning of other parameters except Table 2**
*ϑ* ——the camera visual acuity;
$d_{w}$ —-the theoretical inter-camera distance;
*α* ——the convergence rotation angle;
*μ* ——the shape ratio;
$h_{D}$ —-the viewing distance;
$t_{e}$ ——the viewer’s inter-ocular distance;
d/h —the index of the 1/30 rule;
*ξ* ——the index of binocular overlap percentage;
*g* ——the index of the convergence rotation angle;
*k* ——the index of the camera visual acuity;
*Z* ——the stereo camera type;
*Q* ——the overall quality score.
